# Editorial: Law and neuroscience: justice as a challenge for neurorights, neurolaw, and forensic psychology

**DOI:** 10.3389/fpsyg.2025.1615234

**Published:** 2025-05-19

**Authors:** Eric García-López, Cristina Nombela, Eduardo Demetrio Crespo

**Affiliations:** ^1^Neurolaw and Forensic Neuropsychology, Scientific Research Department, Instituto Nacional de Ciencias Penales, Mexico City, Mexico; ^2^Biological and Health Psychology, Universidad Autónoma de Madrid, Madrid, Spain; ^3^University of Castilla-La Mancha, Toledo, Spain

**Keywords:** law, neuroscience, neurolaw, forensic psychology, neurorights, criminal responsibility

Since the introduction of the term “neurorights” by Ienca and Andorno (2017) and Yuste et al. (2017), there has been a significant increase in academic interest in this concept. The notion of “neurorights” (Ienca, 2021) emerges from translational knowledge that involves various fields, including Law, Philosophy, and Neuroscience. However, this interdisciplinary dialogue faces inherent difficulties due to variations in terminology and methodology across disciplines.

One prominent example is the concept of “free will,” which has traditionally been associated with philosophical and legal debates. Neuroscience research, notably by Libet et al. (1983) and Gruart and Delgado-García, has experimentally explored the neural foundations of voluntary action, raising questions about the very existence of free will (Libet, 1985). Nevertheless, from a cognitive and behavioral perspective, free will encompasses more than motor actions; it involves cognitive processes that are essential in Psychology (Baumeister, 2008; Racine, 2017) and Forensic Psychiatry (Morse, 2007; Meynen, 2009; Schleim, 2012).

Recent advancements in neuroimaging have substantially impacted our understanding of criminal responsibility (Glannon, 2014; Vitacco and Coleman, 2024), specifically with regard to legal concepts such as culpability and dangerousness. These developments pose significant challenges for Forensic Psychology and Psychiatry, raising questions such as: How do these neuroscientific insights reshape our understanding of mental disorders and their forensic implications (Morse, 2015; Meynen, 2013, 2015)? How will violence risk assessments evolve (Haarsma et al., 2020)? How might these insights inform research in victimology, such as the evaluation of torture or gender-based violence (García-López, 2024)? Do criminological models of decision-making in psychiatric contexts require reevaluation (Levander and Levander)?

Furthermore, neurorights are increasingly relevant due to advancements in Neurotechnology and Artificial Intelligence, raising critical human rights concerns that are being addressed by international organizations and government agendas worldwide (UNESCO, 2023; Andorno, 2023; Garrigues Walker and González de la Garza, 2024). Commercial applications, such as those pursued by companies such as Neuralink, necessitate clear regulatory frameworks to mitigate the risks and ethical dilemmas involved (Pérez Manzano, 2023; González Tapia, 2023). Various international bodies have initiated protective measures, exemplified by the Inter-American Declaration of Principles on Neuroscience, Neurotechnologies, and Human Rights (Inter-American Juridical Committee - OAS, 2023), Mexico's proposed General Law on Neurorights (Herrera-Ferrá et al., 2025), and Chile's constitutional reforms (Muñoz, 2019; McCay, 2024), despite ongoing debates (Bublitz, 2023; Ruiz et al., 2024).

This paper aims to highlight two key areas of research: (1) personal identity in terms of cognitive freedom and mental privacy, both as a basis for assigning criminal responsibility and as a fundamental right to be protected; and (2) forensic implications of neurotechnological advancements in the assessment of criminal behavior, particularly with regard to tools for evaluating risk of violence and recidivism. These topics should be examined from neuroscientific, empirical, methodological, and interdisciplinary perspectives. The field of criminal justice is increasingly incorporating predictive neurocognitive methodologies and machine learning algorithms, significantly influencing real-world forensic and judicial decision-making processes (Miró Llinares and Castro Toledo, 2002).

This Research Topic brings together 18 rigorously developed scientific articles addressing critical issues, including the role of neurotechnology in memory neuromodulation (González-Márquez), false memory evaluation (Pérez-Mata and Diges), and potential mind-reading technologies (Andorno and Lavazza). Other central topics include neuroscientific analyses of criminal behavior (Lee), virtual emotions (González-Tapia), and evidence-based sentencing (Martínez-Garay). The Research Topic features original research on juvenile justice systems (Patiz and Bayraktar), sentencing for dangerous driving offenses (Liu et al.), ethical considerations in the integration of computer perception with neurotechnology (Hurley et al.), cognitive strategies in child custody decisions (de Alcântara Mendes and Ormerod), self-control in criminology (Levander and Levander), and bias differences in judicial simulations involving avatars vs. humans (Frumkin et al.).

In summary, this Research Topic underscores the necessity of interdisciplinary collaboration among Neuroscience, Law, and Forensic Psychology to address the profound challenges that neurotechnological advancements pose for justice systems and the broader notion of justice itself.
